# A Primary Screening and Applying of Plant Volatiles as Repellents to Control Whitefly *Bemisia tabaci* (Gennadius) on Tomato

**DOI:** 10.1038/srep22140

**Published:** 2016-02-24

**Authors:** Wenxiao Du, Xiaoqing Han, Yubo Wang, Yuchuan Qin

**Affiliations:** 1 Department of Entomology, China Agricultural University, Beijing 100193, China; 2 Tangshan Academy of Agricultural Sciences, Tangshan City 063001, China; 3 Dry-Land Farming Institute of Hebei Academy of Agricultural and Forestry Sciences, Hengshui 053000, China

## Abstract

With the goal of finding a new way to reduce population densities of *Bemisia tabaci* biotype Q in greenhouses, seven repellent volatile chemicals and their combinations were screened. The mixture of DLCO (D-limonene, citral and olive oil (63:7:30)) had a better cost performance(SC_50_ = 22.59 mg/ml)to repel whiteflies from settling than the other mixtures or single chemicals. In the greenhouse, in both the choice test and the no-choice tests, the number of adult whiteflies that settled on 1% DLCO-treated tomato plants was significantly lower than those settling on the control plants for the different exposure periods (P < 0.01). In the choice test, the egg amount on the treated tomato plants was significantly lower (P < 0.01) than that on the control plants, but there was no significant difference (P > 0.05) between the number of eggs on treated and control plants in the no-choice test. Compared with the controls, 1% DLCO did not cause significantly statistic mortality rates (P > 0.05) out of different living stages of *B. tabaci*. The tests for evaluating the repellent efficacy, showed that a slow-releasing bottle containing the mixture had a period of efficacy of 29 days, and the application of this mixture plus a yellow board used as a push-pull strategy in the greenhouse was also effective.

The whitefly *Bemisia tabaci* (Gennadius) biotype Q (Hemiptera: Aleyrodidae) is a serious pest of tomato plants worldwide. Both adults and larvae cause direct damage by continual sap sucking and by excreting honeydew, which accumulates on the tomato plant, causing chlorosis of infested leaves and the growth of sooty mold. *B. tabaci* also causes substantial indirect damage as an efficient vector of plant pathogenic viruses, primarily Gemini viruses^1^ and the tomato yellow leaf curl virus-Israel (TYLCV-Is)^2^. Members of Geminiviridae interfere with plant photosynthesis, which seriously reduces productivity and product market value (fruit and fiber)^3^.

*B. tabaci* is a species complex that includes many genetically differentiated populations, of which the B and Q biotypes are widely distributed. The Q biotype was first recognized in the south of Spain and Portugal^4^, and many studies have demonstrated that *B. tabaci* biotype Q has a higher tolerance to extreme temperatures and a higher resistance to insecticides than the biotype B^5^,^6^,^7^,^8^,^9^. Biotype Q has a worldwide distribution that includes Morocco^10^, Egypt^11^, Israel^12^, Croatia^13^, Montenegro^14^, Italy^15^ and China^16^. Because of its extensive host range and ability to transmit a relatively large number of plant viruses, the Q biotype is of particular concern^17^. As demonstrated by recent research, whitefly biotype Q has caused severe damages in many regions of China^18^,^19^.

Although chemical control is widely used for the management of *B. tabaci*, whitefly has developed resistance to a number of insecticides^20^. Furthermore, the excessive use of insecticides has resulted in the elimination of natural enemies of whitefly^16^, leading to an ecological crisis in tomato production systems. This situation has led to the development of integrated pest management (IPM) strategies, in which biological control plays a central role^21^.

Various petroleum oils and plant-derived oils have repellent activities against insect pests^22^, demonstrating the potential for their use in IPM. The essential oil of *Citrus limon* (L.) was reported to repel the dengue vector *Aedes albopictus* (Skuse)^23^. The essential oils of *Eucalyptus citriodora* Hook and *Tagetes lucida* Cavanilles. grown in Colombia demonstrated repellency against *Sitophilus zeamais* (Motschulsky) at doses between 0.063 and 0.503 ml/cm^2^ 24. Ginger oil has insect growth regulator and antifeedant activity against *Spilosoma obliqua* (Walker) larvae^25^. The essential oil of oriental arborvitae *Platycladus orientalis* (L.) Franco appears to have potential as a control agent against cowpea weevil (*Callosobruchus maculatus* Fab.) and rice weevil (*Sitophilus oryzae* L.) adults^26^. D-limonene from celery (*Coriandrum sativum* L.) and geranyl nitrile from Malabar spinach (*Basella alba* L.) reduced whitefly colonization significantly^16^. Limonene, extract from celery (*C. sativum* L.), was able to repel whitefly, and used in a “push–pull” strategy to control *B. tabaci*^27^. Citronellal oil and olive oil could reduce settling of whitefly adults^28^. Citral could be used to control *Grapholita molesta* (Lepidoptera: Tortricidae Busck) by mating disruption^29^. α-Pinene had the repellency against stripped flea beetle (*Hyllotrata striolata* F)^30^. Considering the reason of economic and repellent efficiency, some repellent volatile chemicals on above were chosen for this research.

Yellow boards covered with adhesive are widely used as an insect control tool that can attract and kill whiteflies and maintain their populations at tolerable levels for approximately 30 days^31^. However, satisfactory results with yellow boards were only obtained with low densities of whitefly^31^. Nonetheless, combining yellow boards with repellent oils may bolster the pest-reducing capability of yellow boards by employing a ‘push’ and ‘pull’ strategy for better control of *B. tabaci*.

The objectives of this experiment were to test the efficacy of seven volatile chemicals and their mixtures as repellents for *B. tabaci* biotype Q and to identify the most effective chemical or mixture. Additionally, the chemicals were tested for contact toxicity toward *B. tabaci* biotype Q, and the optimal methods of applying monomers or mixtures plus yellow boards as a push-pull strategy in the greenhouse were evaluated.

## Results

### Laboratory bioassays

**Figure 1:**
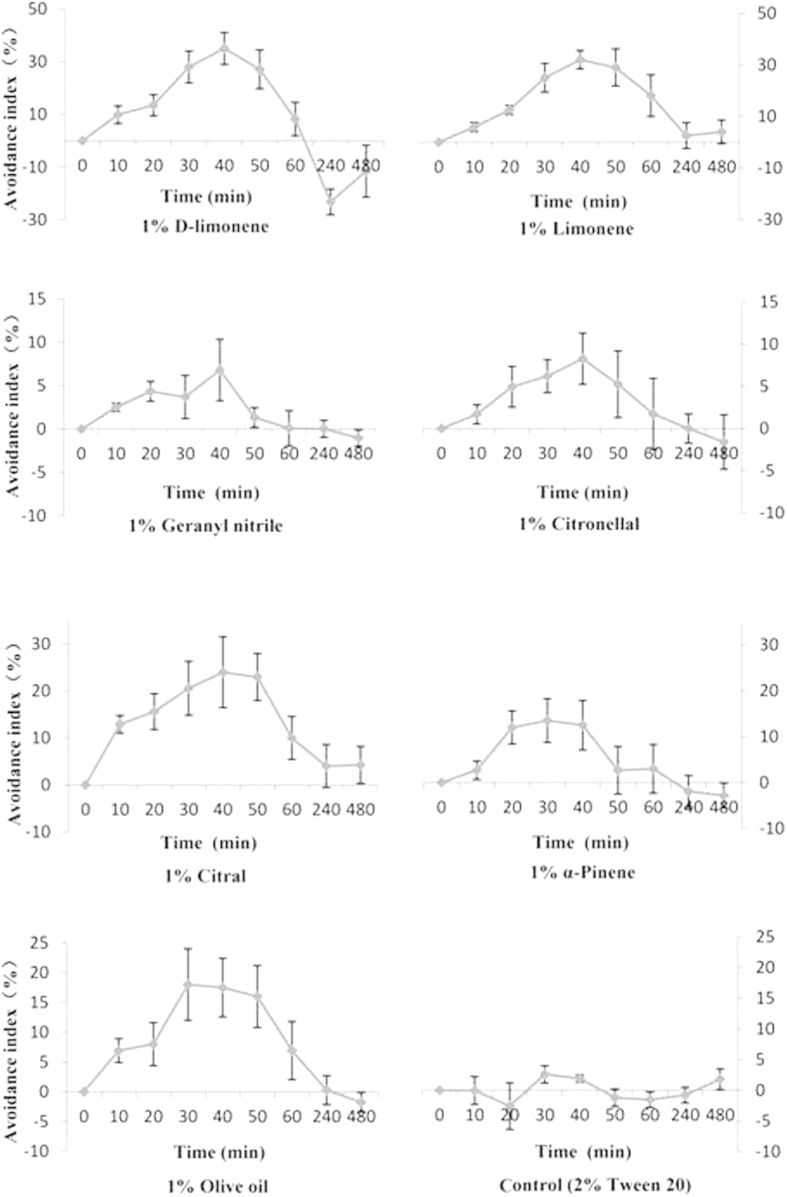
Time-dependent avoidance indices (±SD) of seven plant volatile repellents tested singly and plus control (2% Tween 20).

All seven volatile chemicals caused avoidance in the whitefly, whereas the 2% Tween 20 did not. All seven volatile chemicals caused avoidance within 10 min, and the AIs peaked between 30 and 40 min (Fig. 1).

Of all the volatile chemicals, limonene and citral were similar to D-limonene base upon comparisons of fiducial limits of *SR*_*50*_ (Table 1). D-limonene had a smaller *SR*_*50*_ value than lemonile, citronellal, α-Pinene, and olive oil. The combination of D-limonene and citral with olive oil in the ratio of 63:7:30 and 81:9:10 had a smaller *SR*_*50*_ values than D-limonene, but for economic reasons (as a kind of dietary fat^32^, olive oil had the lowest price among all the chemicals used in this experiment), the ratio of 63:7:30 was chosen for subsequent experiments.

### Toxicity toward different developmental stages

Compared with the controls, 1% DLCO did not cause significantly statistic mortality rates (P > 0.05) when eggs (F = 0.77; df = 2, 6; P = 0.51), larval stages (F = 1.31; df = 2, 6; P = 0.34), pupae (F = 0.44; df = 2, 6; P = 0.66) and adults (F = 2.30; df = 2, 6; P = 0.18) were treated.

### Choice and no-choice tests

**Figure 2:**
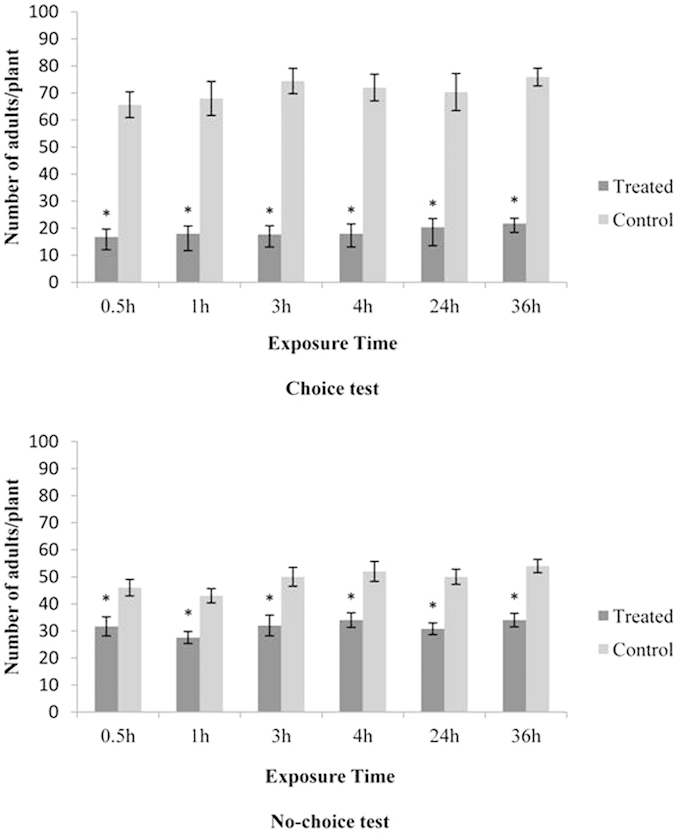
Mean number (±SEM) of whitefly adults on treated (1% DLCO) and control (2% Tween 20) tomato plants at 0.5, 1, 2, 3, 4, 24 and 36 h after release in a choice test and no-choice test conducted in the greenhouse. Different letters above bars indicate significant differences among treatments (t-test: P ≤ 0.05).

In the choice test, the number of adult whiteflies that settled on 1% DLCO-treated tomato plants was significantly lower than those settling on the control plants for the different exposure periods (T-test, P < 0.01) (Fig. 2). The mean number of adult whiteflies on the treated plants ranged from 16.8 to 21.7 per plant, which was 55.5–61.5% lower than the number on control plants (ranged from 65.7 to 75.9) (Fig. 2). After 36 h, the number of eggs laid on the treated tomato plants (71.6 ± 9.8) was 57% lower than that on the control plants (264.2 ± 20.0) (t = −24.44; df = 4; P < 0.01).

In the no-choice tests, the number of adult whiteflies on tomato plants treated with 1% DLCO ranged from 27.6 to 34.0, which was 18.4 to 23.8% lower than the number on control plants for the different exposure periods (T-test, P < 0.01) (Fig. 5). After 36 h, no significant difference between the number of eggs on treated (275.4 ± 39.4) and control plants (265.1 ± 34.4) was found (t = −0.45; df = 4; P = 0.68).

### Repellent effect

Two methods (tomato plants sprayed with 1% DLCO or slow-releasing bottles containing 1% DLCO hung over tomato plants) were compared, and a 3-way ANOVA was used to do the statistics from the data of treatment, distance, time and interactions. Results showed that treatment, distance, time and interactions affected the number of adult whiteflies significantly (P < 0.01) (Table 2).

**Figure 3:**
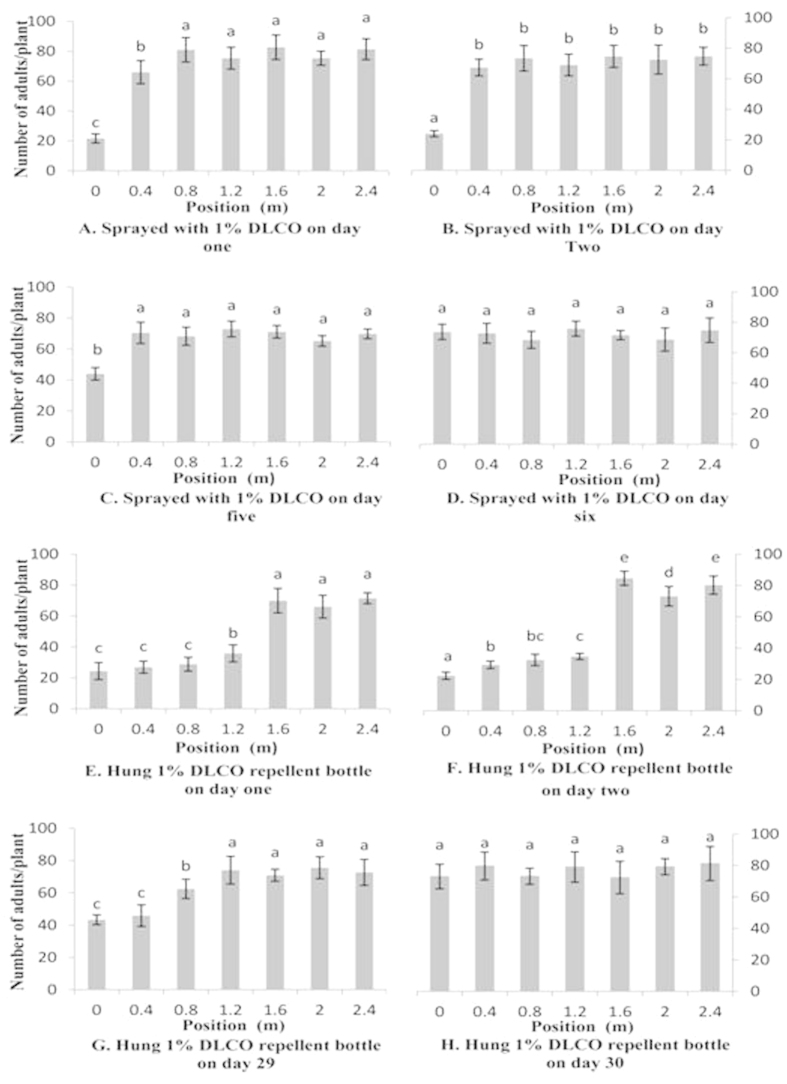
Mean (±SEM) number of whitefly adults distributed in two treatments. Different letters on top of the bars indicate significant differences among the plants (Tukey’s HSD’s test: P ≤ 0.05).

In the spraying treatment, there were significantly differences between number of adults on plants at different distances from day one to day five (P < 0.01). The number of adult whiteflies on the center tomato plant (0 m) was significantly reduced on the first day comparing with the other plants at different distances (F = 89.6; df = 6, 14; P < 0.01) (Fig. 3A). More whiteflies settled on tomato plants at 0.4 m than on the center plants (Fig. 3A), but there were no significant differences between the number of whiteflies on tomato plants located at distances further than 0.4 m on the first day (Fig. 3A). On the second day, there were still significant differences between the number of adult whiteflies on the center tomato plant (0 m) and other tomato plants at different distances (F = 56.66; df = 6, 14; P < 0.01) (Fig. 3B). The number of adults on plants at 0.4 m was not significantly different from that on plants at 0.8 m on the second day (Fig. 3B). Differences were not detected between distances on the sixth day (F = 2.47; df = 6, 14; P = 0.08) (Fig. 3D). Thus, the spraying treatment had an effective repellent range of approximately 0.4 m from the center for five days.

With the slow-releasing bottles, the numbers of whitefly adults that settled on plants at different distances had been keeping significant differences from the first day to day 29 (P < 0.01). On the first day the adult whiteflies settled more on tomato plants at 1.2 m than on the center plants (F = 129.03; df = 6, 14; P < 0.01), but there were no significant differences between the number of whiteflies on tomato plants located at distances further than 1.2 m (Fig. 3E). Number of whiteflies on tomato plants was significantly different on day two (F = 880.75; df = 6, 14; P < 0.01) (Fig. 3F). At day 29, there were no significant differences between 1.2 m and more distant plants. After 30 days, all the plants had a similar number of adult whiteflies (F = 1.69; df = 6, 14; P = 0.20) (Fig. 3H). Thus, the slow-releasing bottle had an effective repellent range of 0.8–1.2 m from the center, and the period of repellence was approximately 29 days.

### Push-pull strategy application

The population densities of adult whiteflies on tomato plants were significantly lower in the two treatments than in the control and were also significantly different between the two treatments (F = 38.48; df = 2, 6; P < 0.01). The average number of adults on each plant in treatment one (slow-releasing bottles containing 1% DLCO plus yellow boards hung over tomato plants) was 17.5 ± 3.8, whereas it was 29.1 ± 2.7 for treatment two (yellow boards only) and 47.0 ± 4.5 for the control plot.

## Discussion

Previous research has indicated that ginger oil can protect tomato seedlings from whitefly settling and oviposition, and this repellency of ginger oil was attributed to its odor^33^. The research of Sertkaya (2010) showed that essential oils obtained from medicinal plants such as thyme (*Thymbra spicata subsp. spicata* L.), fennel (*Foeniculum vulgare* Mill.) and rosemary (*Rosmarinus officinalis* L.) were repellent to cotton whitefly adults^34^. Although not all of the tested volatile chemicals in the present study have been previously investigated with regard to repellent and deterrent activities against *B. tabaci*, the potential of most of them to be repellents of other insects is known. For example, the oils of lemongrass (*Cymbopogon citratus* L) and citronella (*Cymbopogon winteriana* L) produced strong irritant and repellent responses in *Aedes aegypti* (Diptera: Culicidae)^35^, and *Cymbopogon spreng* (L.) essential oil repelled *Myzus persicae* (Hemiptera: Aphididae) on field tobacco^36^. Limonene diluted 500 times had a 62% greater deterrent effect on adults than controls, repelling whitefly egg-laying by more than 80% in the greenhouse^27^. D-limonene and geranyl nitrile were reported to reduce whiteflies on cucumber^16^. In contrast, olive oil, was less effective in reducing adult whitefly settling than other compounds^28^, but the combination of D-limonene, citral and olive oil was effective with a ratio of 63:7:30 in the current study.

The direct airborne repellent assay, which was improved, was used in this experiment to identify and evaluate a relatively suitable volatile chemical mixture, DLCO. It has the advantage of direct repulsion to an airborne chemical vapor and less time required for pest control comparing with trap assays (a former way to test the repellent effect)^37^,^38^. However, the assay monitors only the airborne vapor of materials rather than a contact chemical response because the screen net separates the adult whiteflies. Although the toxicity bioassays of the mixture toward eggs, larvae, pupae and adults showed that the compounds were safe for the whitefly, additional toxicity and repellency tests are necessary.

Phytotoxicity is likely to be a problem in the application of volatile chemicals to tomato plants. Indeed, cotton seed oil sprayed directly over the top of plants caused severe damage^39^, and the concentration of oil spray droplets on the youngest developing leaves resulted in injury. In this greenhouse research, a few necrotic spots appeared on some of the treated leaves at concentrations above 1%, while at concentrations up to 10%, severe wilting and death of the entire treated plant occurred. For this reason, the 1% concentration was used in the toxicity bioassays and the greenhouse choice and no-choice tests.

The greenhouse choice and no-choice tests indicated that spraying tomato plants with the screened mixture effectively reduced the settling of adult whiteflies. Although both sides of the leaves were covered thoroughly by spraying, the deposit was too thin, and evaporation could not be controlled. Because of gravity, the volatile chemical mixture on the adaxial surface of the tomato leaves was not retained as much as that on the abaxial surface. Thus, a way of retaining the volatile chemical mixture for a longer time on the plant might be necessary, particularly because evaporation tends to be higher in the open field. By comparison, the application with the slow-releasing bottle showed better coverage, and the bottle application was synergistic when combined to yellow boards.

In conclusion, this study describes a new repellent with potential for control of whiteflies. Following the approach described here, more effective avoidance agents can be identified and applied for the control of whiteflies.

## Methods

### Plant materials

‘Hybrid 101’ tomato (*Solanum lycopersicum* L.) seeds provided by the Chinese Academy of Agricultural Sciences were sown in a greenhouse in plastic pots (10 cm in diameter). The plants were grown inside net cages (120 mesh gauze) to exclude insects. The greenhouse had natural light and an average temperature of 27 ± 4 °C.

### Insects

*B. tabaci* (biotype Q), which was used in all bioassays, was collected in June 2010 from tomato fields in the southern suburbs of Beijing, China (116.2°E, 39.5°N). The insects were reared in isolation on tomato plants under standard laboratory conditions (26 ± 2 °C, 60–70% RH and a photoperiod of 14:10 h (L:D)), and the whiteflies were not exposed to any insecticides. The identification of whiteflies (biotype Q of *B. tabaci*) was performed according to RAPD-PCR^40^.

### Laboratory bioassays

**Figure 4:**
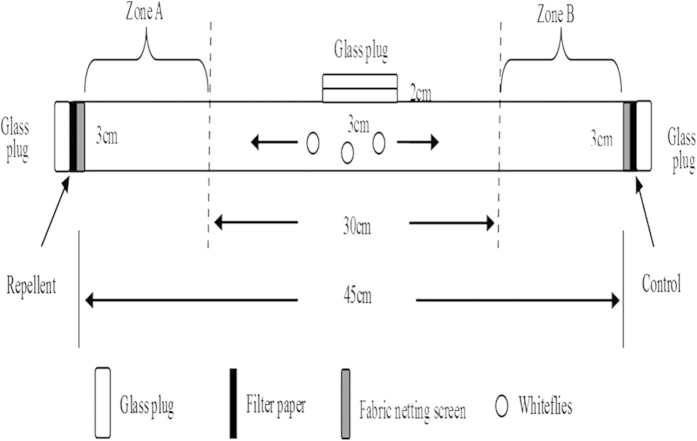
The apparatus used in bioassays. Before the assay, a volatile chemical (treatment) was applied to the filter paper between the coarse fabric netting screen and the bottom of the assembly, and then the glass plug was covered. The opposite plug was used as the control. Zone A was the treatment area, and zone B was the control area.

The commercially volatile chemicals used in this study are described in Table 3. The bioassays were conducted from 8:00 a.m. to 6:00 p.m. in a temperature-controlled room at 27 °C and 40% RH. A binary-choice bioassay tube, which was considered as a specific performance of direct airborne repellent assay, was used to measure repulsion to volatile chemicals (Fig. 4). The bioassay arena was constructed of clear glass (45 cm in length by 3 cm in diameter) with three stoppers (left, right, and upper). The lateral stoppers were covered with a 2.5-cm-diameter filter paper liner circle with coarse fabric netting outside to prevent the whiteflies from direct contact with the chemicals (Fig. 4). Twenty microliters of volatile chemical (vol:vol, containing 2% Tween 20 in deionized water) was added to one end (left or right glass plug) of the filter paper, and a control (deionized water) was added to the other end of the test tube. One hundred *B. tabaci* adults of unknown sex and age were introduced into the tube from the middle hole (upper glass plug).

First, seven chemicals (1%, vol:vol) and 2% Tween 20 in deionized water were tested to identify a suitable evaluation time. After the incubation period (10 min, 20 min, 30 min, 40 min, 50 min, 60 min, 240 min and 480 min), the whiteflies were counted in the two test zones, and the avoidance index (AI)^37^ was calculated:


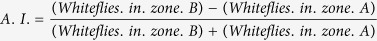


where zone A was the treatment area, and zone B was the control area. Thus, a time-dependent AI was found for each volatile chemical. Trials were repeated three times for each volatile chemical test.

To identify suitable concentrations of the volatile chemicals, a linear regression was used with two variables *AI* (y) and concentration (x). In this test, the seven volatile chemicals and their mixtures were tested at six concentrations (0.01%, 0.1%, 1.0%, 10%, 50%, and 70%, vol:vol, containing 2% Tween 20 in deionized water), and there were three replications for each concentration; deionized water alone served as the control. For each volatile chemical, the concentration was determined at which 50% of the whiteflies avoided settling (*SC*_50_). The *SR*_50_ values come from each *SC*_*50*_value of the chemicals divided by D-Limonene’s. The binary-choice bioassay tube was washed with soap and water, rinsed with 90% ethanol and dried between experiments.

### Toxicity toward developmental stages

Egg, larva and pupa bioassays. Tomato plants (see above) with three leaves were placed in a net cage (35 cm × 35 cm × 35 cm, 120 mesh gauze) and inoculated with 300 adult whiteflies for egg, larva and pupa bioassays. Tomato leaves infested with 0–2-day-old eggs, larvae or pupae were sprayed with 4 ml per leaf of a 1% volatile chemical mixture (D-limonene: citral: olive oil at 63:7:30) with 2% Tween 20 (referred to henceforth as 1% DLCO), deionized water (control 1), or 2% Tween 20 diluent (control 2). The cumulative mortality of eggs and larvae had been recording for 18 days after the application; the cumulative pupal mortality had been recording for 10 days after the pupae emerged. One tomato plant was treated and checked for each treatment for each bioassay, and all bioassays were repeated three times with totally 27 different net cages and 27 different plants^35^.

Adult bioassays. A single tomato plant (free of whiteflies) with three leaves was sprayed with 4 ml of 1% DLCO, deionized water (control 1), or 2% Tween 20 diluent (control 2). After air drying, each of the treated tomato plant was placed respectively in one net cage (35 cm × 35 cm × 35 cm, 120 mesh gauze), and 300 adult whiteflies were introduced into each net cage. Adult mortality was recorded after 7 days. The experiment was repeated three times with a total of 9 different net cages and 9 different plants^35^.

### Choice and no-choice tests

Treated and control tomato plants with three leaves were used as hosts for whiteflies and air dried at room temperature for 1 h before use^33^.

Choice tests. Thirty-two tomato plants were placed in one net cage (7 m × 2 m × 1.5 m, 120 mesh gauze) in the greenhouse and divided evenly into two groups: a treatment group sprayed with 1% DLCO and a control group sprayed with 2% Tween 20. The distance between each tomato plant was 40 cm in each group, and the distance between the two groups was 4 m^41^. 1800 adult whiteflies were released into the center of the cage. The whiteflies that settled on both sides of the leaf of each tomato plant were counted at 0.5, 1, 2, 3, 4, 24, and 36 h after release. The eggs on both sides of the leaf were counted under a dissecting microscope at 20 x magnification 36 h after the whiteflies were released. The experiment was repeated three times with different plants.

No-choice tests. Tomato plants were placed in net cages (35 cm × 35 cm × 35 cm, 120 mesh gauze) in the greenhouse; each cage contained one tomato plant. Two cages were used for the treatments, and two cages were used for controls. The treated plants were sprayed with 1% DLCO, and the control plants were sprayed with 2% Tween in distilled water. The cages were assigned at random in the greenhouse, and 60 adult whiteflies were released into each cage. On both sides of the leaf of each tomato plant the adults were counted at 0.5, 1, 2, 3, 4, 24, and 36 h after release by direct observation, the eggs were counted under a dissecting microscope at 20 x magnification 36 h after the whiteflies were released. The experiment was repeated three times on three alternate days with different plants.

Avoidance indexes (AIs) were calculated according to the number of adults or eggs in the treatments and controls in the choice and no-choice tests.

### Repellent effect

**Figure 5:**
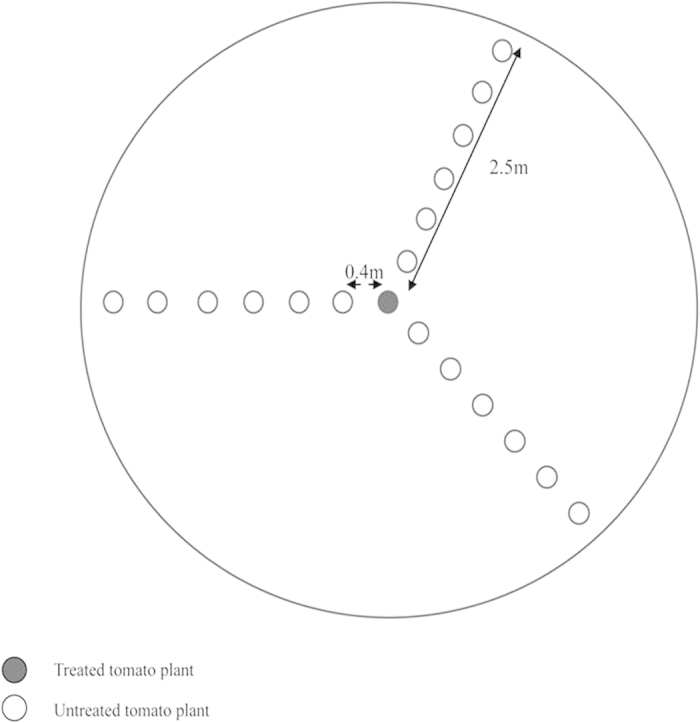
Design of circular arena for the repellent effect determined by two methods (tomato plant sprayed with 1% DLCO or slow-releasing bottle containing 1% DLCO hung over tomato plant).

Nineteen tomato plants were placed in one circular arena (5 m diameter × 1.5 m height, 120 mesh gauze) which was used for each treatment or control (Fig. 5). The distance between adjacent tomato plants was 0.4 m. For treatment one, a tomato plant was sprayed with 4 ml of 1% DLCO at the center of the arena; for treatment two, one slow-releasing bottle with 16 ml DLCO hung on the center plant (The bottle is made of polymer material, repellents can across the molecular gap of the polymer material slowly, which can extend the period of repellents. The bottle volume for the experiment is 16 ml).

Approximately 1800 adult whiteflies were released into the circular arena for each treatment or control, and the whiteflies were counted daily after release on each plant. The ranges and lengths of time of the repellent effect of each volatile chemicals were determined^42^. The experiments were repeated three times with different plants.

### Push-pull strategy application

**Figure 6:**
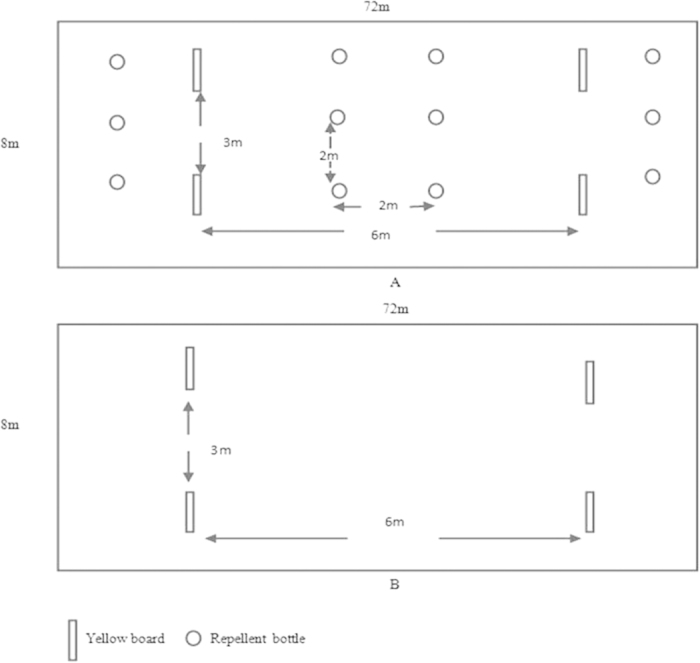
Design of field trials in greenhouse.

The greenhouses (72 m × 10 m × 2.5 m) were located in Tangshan, Hebei Province, China (118.11°E, 39.36°N). The row spacing of the tomato plants was 80 cm, and the plant spacing within rows was 40 cm. Two treatment plots (treatment one, slow-releasing bottles (1% DLCO) plus yellow boards hung over tomato plants; treatment two, only yellow boards hung over tomato plants) and one control plot (no slow releasing bottles or yellow boards) were used, and each of these was located in a separate room (72 m × 8 m; Fig. 6). The distance between the bottles was within double the repellent’s effective range (2 m) (Fig. 6). The door of each room was open to allow wild whiteflies to enter. The greenhouse tests were repeated three times with different plants.

The adult whiteflies that settled on the tomato plants in the treatment and control plots were counted every five days using a five-point sampling method^42^. For each sampling point, six leaves (three located in the upper layer of the plant and three in the bottom layer) were checked to record adult whiteflies. The total number of sampled leaves was 720 for each treatment (average of 4 weeks of sampling) in the greenhouse.

### Statistical analyses

Linear regression, paired T-tests, ANOVAs followed by Tukey HSD (Honestly Significant Difference) tests were used to analyze the number of whitefly behavioral responses to volatile chemicals in the laboratory or greenhouse bioassays^16^. All statistical analyses were performed with SPSS (version 16.0 for Windows, SPSS Inc., Chicago, IL, USA) or PoloPlus (version 2.0 for Windows, LeOra Software).

## Additional Information

**How to cite this article**: Du, W. *et al*. A Primary Screening and Applying of Plant Volatiles as Repellents to Control Whitefly *Bemisia tabaci* (Gennadius) on Tomato. *Sci. Rep*. **6**, 22140; doi: 10.1038/srep22140 (2016).

## Figures and Tables

**Table 1:** Effect of various compounds on the settling of *B. tabaci* adults on tomato plants.

Material	Mixing ratio^a^	Intercept	Slope	*SC*_50_^b^ (95% confidence limits) (mg/ml)	*SR*_50_^c^	P^d^
D-Limonene	–	−0.81	0.41	88.33 (43.15–215.64)	1.0	0.79
Limonene	–	−1.11	0.54	115.76 (63.20–243.15)	1.30(0.46–3.69)	0.70
Geranyl nitrile	–	−2.55	0.94	513.60 (370.54–769.80)	5.81(2.43–13.92)	0.77
Citronellal	–	−3.04	1.09	614.14 (463.08–907.33)	6.95(2.95–16.37)	0.92
Citral	–	−1.42	0.61	215.40 (120.03–449.77)	2.45(0.79–7.58)	0.76
α-Pinene	–	−1.85	0.70	420.99 (274.46–685.40)	4.76(1.90–11.93)	0.87
Olive oil	–	−1.44	0.43	2110.40 (881.47–3377.84)	23.89(6.46–88.33)	0.31
D-Limonene:Citral	1:9	−5.40	2.48	149.62 (129.18–169.23)	1.69(0.76–3.79)	0.61
	3:7	−4.40	2.13	117.25 (92.64–140.88)	1.30(0.85–4.43)	0.42
	5:5	−3.46	1.77	91.02 (65.48–114.88)	1.03(0.44–2.39)	0.23
	7:3	−3.35.	1.82	69.46 (38.23–98.14)	0.79(0.32–1.95)	0.82
	9:1	−4.87	2.81	53.84 (41.84–65.86)	0.61(0.27–1.42)	0.24
D-Limonene:Citral:Olive oil	9:1:90	−3.37	1.67	107.66 (87.61–146.61)	1.21(0.52–2.80)	0.28
	27:3:70	−3.02	1.57	82.39 (69.21–104.06)	0.55(0.25–1.22)	0.27
	40:5:50	−4.04	2.27	58.74 (50.39–70.17)	0.67(0.29–1.49)	0.67
	63:7:30	−0.69	0.51	22.59 (12.98–33.77)	0.25(0.10–0.63)	0.90
	81:9:10	−1.15	0.74	36.00 (24.85–52.51)	0.4(0.18–0.90)	0.57

^a^The ratio is vol:vol.

^b^*SC*_*50*_ = estimated concentration of tested volatile chemicals required to reduce adult settling by 50%, and the value is the mean of four *SC*_*50*_’s.

^c^Setting rates based upon *SC*_*50*_values relative to D-Limonene as the standard. *SR*_50_ comes from each *SC*_*50*_value of the chemicals divided by D-Limonene’s.

^d^P > 0.05 indicates that the data fit the Probit model.

**Table 2:** The result of 3-way ANOVA with treatment, distance, time and interactions.

Source	df	F value	P
Time	29	41.83	<0.01
Distance	6	998.68	<0.01
Treatment × Time	58	17.93	<0.01
Treatment × Distance	12	769.32	<0.01
Time × Distance	174	7.15	<0.01
Treatment × Time × Distance	348	5.59	<0.01
Error	1260		

**Table 3:** Compounds used in bioassays.

Common name	Chemical registration number	Color	Molecular formula	Product	Purity	Company
D-Limonene	5989-27-5	Colorless	C10H16	Lemongrass oil, pine needle oil, vanilla oil, turpentine	95%	Codow
Limonene	138-86-3	Orange-red, orange-yellow, or colorless	C10H16	Citrus	95%	Codow
Geranyl nitrile	5146-66-7	Faint yellow or colorless	C10H15N	Lemon oil	97%	Codow
Citronellal	106-23-0	Faint yellow or colorless	C10H20O	Citronellal oil, eucalyptus oil	96%	Codow
Citral	5392-40-5	Faint yellow or colorless	C10H16O	Lemongrass oil, lemon oil, orange oil, Litsea cubeba oil, verbena oil, basil oil	97%	Codow
α-Pinene	80-56-8	Colorless	C10H16	Turpentine	98%	Sigma
Olive oil	8001-25-0	Golden yellow	C51H92O6	Olives	≥99.5%	Codow
